# Radiation Therapy Skin Marking with Lancets Versus Electric Marking Pen (COMFORTATTOO)—6 Months Results on Cosmesis, Fading, and Patients’ Satisfaction From a Randomized, Double-Blind Trial

**DOI:** 10.1016/j.adro.2023.101404

**Published:** 2023-11-05

**Authors:** André M. Pires, Luísa Carvalho, Ana C. Santos, Ana M. Vilaça, Ana R. Coelho, Celeste Oliveira, Céline Costa, Flávia Fernandes, Liliana Moreira, João Lima, Rafaela Vieira, Maria J. Ferraz, Marta Silva, Pedro Silva, Rafael Matias, Sara Zorro, Susana Costa, Susana Sarandão, Ana F. Barros

**Affiliations:** Radiation Oncology Department, Portuguese Institute of Oncology of Porto, R. Dr António Bernardino de Almeida, Porto, Portugal

## Abstract

**Purpose:**

Most of radiation oncology centers rely on set-up skin markings for patient setup during treatment delivery. Permanent dark-ink tattooing is the most popular marking method. COMFORTATTOO is a unicentric, randomized trial testing 2 permanent methods: lancets against an electric marking pen (Comfort Marker 2.0, CM). One substudy was undertaken to test if using the CM translates into a cosmesis, fading, or satisfaction benefit compared with the lancets.

**Methods and Materials:**

Patients aged 18 years or older referred to our department to receive RT were recruited. They were randomly assigned, in a 1:1 ratio, to receive set-up markings using lancets or CM. This substudy aimed to recruit all the living participants included in the main study. The primary endpoints were tattoos cosmesis, tattoos fading, and patients’ satisfaction 6 months after finishing the RT. Cosmetic and fading assessments were scored on a 5-point ascending scale and patients’ satisfaction on a 10-point ascending scale. The trial is registered at ClinicalTrials.gov (number NCT05371795).

**Results:**

Between April and September 2022, 92 patients were enrolled (45 assigned to lancets and 47 to CM) and assessed for the outcomes. Patients receiving CM had significantly better cosmetic markings, with a median score of 4.4 (vs 3.7 for lancets, *P*<.001). On the fading assessment, the CM was associated with lower scores compared with the lancets (median score of 1.3 and 3.3, respectively; *P*<.001). No differences in patients’ satisfaction were observed with either method (median score of 10 for both arms, *P*=.952).

**Conclusions:**

Our substudy results demonstrated that, 6 months after the end of RT, the CM produces better cosmetic markings with less fading compared with the lancets. These differences didn't translate into patients’ satisfaction superiority toward any method.

## Introduction

In radiation therapy (RT), set-up skin markings are used in several radiation oncology centers for target localization, to ensure set-up reproducibility and accuracy of treatment delivery.^1^^,^^2^ Permanent dark-ink tattooing is the most popular method across most RT departments,^3^ and most often relies on disposable lancets and India ink.^2^ Recently, an electric set-up marking device was developed by Medical Precision B.V, named Comfort Marker 2.0 (CM). It provides controlled depth pigment deposition on the patient skin, which could be advantageous in terms of pain and fading. However, no data comparing these 2 systems are available in the literature, which led to the development of the COMFORTATTOO trial to accurately evaluate the advantages of one system to the detriment of the other.

COMFORTATTOO is a prospective, unicentric, randomized, 2-group cohort study, aimed to establish whether tattooing set-up skin markings using the CM translates into a benefit compared with using disposable lancets. It is the first randomized trial to compare set-up skin tattooing with those specific methods. The coprimary endpoints for the main trial were patient comfort and effectiveness. Secondary endpoints included radiation therapists (RTT) satisfaction and cosmesis.

The issue with dark-ink tattooing is that it creates permanent markings, which act as daily reminders for cancer survivors of their disease and the treatment process. Lancets and CM produce visually distinct tattoos, thus might have some implications concerning cosmesis and fading in the months after finishing the RT. This is particularly important, as an acceptable cosmetic outcome affects the patient experience and satisfaction regarding the oncological treatment.^4^^,^^5^

To assess for cosmesis, fading of the set-up markings, and patients’ satisfaction 6 months following the end of RT, a substudy was undertaken, which results are presented here.

## Methods

### Study Design and Participants

This was a prospective, unicentric, randomized, controlled, parallel, cohort study conducted at a radiation therapy department in northern Portugal.

Eligible patients were adults aged 18 years old or older, referred to our department to receive external beam RT, with an Eastern Cooperative Oncology Group performance status of 0 to 1. An estimated fractionating schedule of at least 13 once-daily fractions was mandatory. Patients requiring either immobilization thermoplastic masks (for head or head and shoulders) or vacuum cushion were excluded. No limit on the maximum number of cutaneous reference points was specified.

All patients provided written informed consent. The study was approved by our local ethics committees (CES 212/021) and the study was performed in accordance with the International Conference on Harmonization Guidelines on Good Clinical Practice and the Declaration of Helsinki.

### Randomization and blinding

In the main study, patients were randomly assigned in a 1:1 ratio to receive skin set-up markings either using disposable lancets (control group) or Comfort Marker 2.0 (experimental group). Eligible patients were randomized via computer-generated random permuted blocks (block sizes of 10), stratified by the number of set-up markings (≤ 4 vs >4). Treatment allocation was blinded to the patients and the radiation therapists (RTTs) presented in the treating rooms but was not possible for the RTTs presented in the CT simulation rooms.

### CT simulation and Set-up skin marking

The set-up markings were created during the simulation session, where a CT simulation was acquired. Once the CT was obtained, the final number of set-up markings required was established, and patients were randomized and received the skin markings with the allocated method. Patients’ marking arrangements were standardized according to the irradiated area and followed our departmental protocols. Typically, patients received 4 to 5 cutaneous reference points, except for the patients who irradiate breast or chest wall who received 9 (for free-breathing techniques) or 11 (for deep-inspiration techniques) reference points.

For the control group, patients’ markings were tattooed using a 28-gauge disposable lancet and India ink. For the experimental group, patients were tattooed with the Comfort Marker 2.0 and the brand black pigment, designed by Medical Precision B.V., using the 0.2 mm depth applications. The patients’ markings were performed by a team of RTTs specifically allocated to the CT simulation, all trained certified to use both methods.

### Outcomes and Assessments

The primary endpoints were tattoos cosmesis, tattoos fading, and patients’ satisfaction 6 months after finishing the RT. A photographic assessment was performed 6 months after the RT was finished. For the cosmetic assessment, all the photographs were scored by 20 observers (both physicians and RTTs) on a 5-point scale (corresponding from bad to excellent cosmesis). For the fading assessment, all the photographs were scored by 3 observers (physicians) on a 5-point scale (corresponding from no fading to complete fading). All the observers were blind to patient identity and treatment allocation. A scheme of the scale used in the fading assessment is provided in the Supplementary Appendix (Fig. E1). For the main study, a similar photographic record was performed at the end of the RT course, which was evaluated for cosmesis and fading using the described methods, allowing a comparison with the scores after 6 months.

The patient satisfaction was assessed on a 10-point numeric scale, with 1 being “completely dissatisfied” and 10 being “completely satisfied.” Additionally, for patients who scored 6 or less, the reason for the discontent was registered.

### Statistical methods

Continuous variables were described by their median, minimum and maximum. Categorical variables were expressed as actual numbers (n) and percentages (%). Normal distribution was checked using Kolmogorov-Smirnov and Shapiro-Wilk tests. Nonparametric tests were applied. Differences between groups were evaluated using Mann–Whitney U test for independent samples and Wilcoxon Signed Ranks test for related samples when comparing continuous variables, and Chi-squared or Fisher's exact tests when comparing categorical variables. Relative risks were calculated on the significant variables.

All tests of statistical significance were 2-sided, and a *P* value <.05 was considered significant. Data were analyzed using IMB SPSS Statistics software, version 28.

The authors are solely responsible for the design and conduct of this study, all study analyses, and the drafting and editing of the manuscript and its final contents.

The trial is registered at ClinicalTrials.gov, number NCT05371795.

This substudy aimed to recruit all the living participants included in the main study.

### Role of the funding source

This work was supported by CIVCO Radiation therapy/Medical Precision B.V, The funder of the study had no role in study design, data collection, data analysis, data interpretation, or writing of the report.

## Results

**Figure 1:**
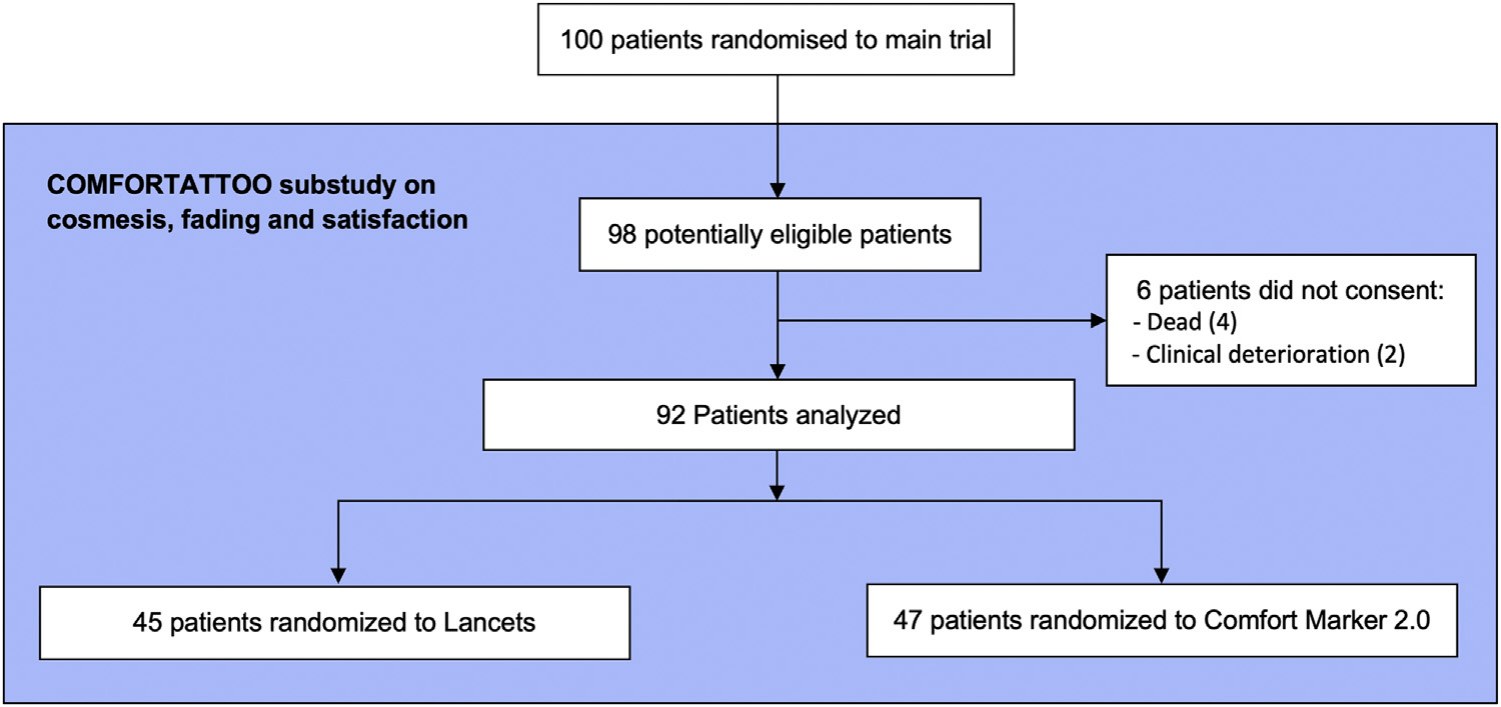
Flow diagram of participation in the cosmesis, fading and satisfaction substudy.

One hundred patients were recruited for the main study between October 2021 and January 2022. Two patients were withdrawn, thus a total of 98 patients were eligible for the substudy. Recruitment opened in April 2022 and closed in September 2022. Six patients weren't enrolled in this analysis (4 were dead before study evaluation and 2 had clinical deterioration), so a total of 92 patients were included (45 patients assigned to lancets and 47 to CM) and analyzed for the outcomes (Fig. 1).

Baseline characteristics were well balanced between the 2 groups (Table 1). The median age of all patients was 59.5 years, and the majority were women (73.9%). The number of set-up markings was also well balanced: 67.4% of the patients in the lancets group and 66.7% of those in the CM group had received >4 set-up markings, with a median of 9 in both groups. Most of the patients included were referred to irradiate breast or chest wall (63.0%), followed by pelvis (22.8%) and thorax (8.7%). There was no significant difference in time between tattooing and the first or the second photographic evaluations between the 2 groups. Characteristics data were comparable to those collected from all patients in the main trial (data not shown).

**Table 1:** Baseline demographic and clinical characteristics at randomization

Variable	Total (N = 92)	Lancets (N = 45)	CM (N = 47)	*P* value
Age, years				
Median (range)	59.5 (25-85)	62 (34-85)	57 (25-78)	.197
Sex, n (%)				
Female	68 (73.9)	31 (68.9)	37 (78.7)	.403
Male	24 (26.1)	14 (31.1)	10 (21.3)	
Irradiated area, n (%)				
Breast/Chest wall	58 (63.0)	26 (57.8)	32 (68.1)	
Pelvis (± perineum)	21 (22.8)	9 (20.0)	12 (25.5)	.186
Thorax	8 (8.7)	6 (13.3)	2 (4.3)	
Other	5 (5.4)	4 (8.9)	1 (2.1)	
Nr of set-up markings				
Median (range)	9 (4-11)	9 (4-11)	9 (4-11)	.383
Nr category, n (%)				
≤4	30 (32.6)	15 (33.3)	15 (31.9)	1.000
>4	62 (67.4)	30 (66.7)	32 (68.1)	
Period between CTsim and 1st photo,* months Median (range)	1.7 (0.9-2.8)	1.7 (0.9-2.8)	1.6 (1.2-2.6)	.766
Period between CTsim and 2nd photo,† months Median (range)	7.6 (5.8-10.0)	7.6 (6.3-10.0)	7.5 (5.8-9.1)	.069

*Abbreviations:* CTsim = computerized tomography simulation; RT = radiation therapy.

⁎ Photos taken on one of the last 3 fractions of RT.

† Photos taken 6 months after the last fraction of RT.

### Cosmesis

The mean scores attributed by the 20 observers for each patient were used for the analysis. Patients receiving CM had significantly higher scores on the photographic assessment (Table 2), with a median score of 3.7 and 4.4 for the lancets and the CM group, respectively (*P*<.001). The proportion of patients who received CM with a mean score of at least 4 was 4.12 times higher than the ones receiving lancets (91.5% vs 22.2%; *P*<.001). The distribution of the cosmesis scores is provided in the Supplementary Appendix (Fig. E2).

**Table 2:** Transversal analysis of cosmesis assessments by study arm

	Lancets (N = 45)	CM (N = 47)	*P* value	RR (CI 95%)
Median (range)	3.7 (1.8-4.4)	4.4 (3.9-4.9)	**<.001**	
Nr category, n (%)				
<4	35 (77.8)	4 (8.5)	**<.001**	
≥4	10 (22.2)	43 (91.5)		4.12 (95% CI, 2.37-7.14)*

*Abbreviations:* CM, comfort maker; RR, relative risk.

⁎ RR for cosmesis ≥4. Median was calculated on the mean scores attributed by the 20 observers for each patient. Lancet was the reference group when the RR was calculated.

Comparing the scores at the end of RT versus after 6 months, no difference was found either for lancets (median of 3.5 vs 3.7, *P*=.061) or CM (median of 4.4 vs 4.4, *P*=.304).

### Fading

The mean scores given by the 3 observers were used for the analysis. The lancets were associated with significantly higher fading scores compared with the CM (median score of 3.3 and 1.3, respectively; *P*<.001; Table 3). While 73.3% of the patients in the lancets group had a mean score of more than 3, in the CM group no patient scored more than 3 (*P*<.001). The distribution of the fading scores is provided in the Supplementary Appendix (Fig. E3).

**Table 3:** Transversal analysis of fading assessments by study arm

	Lancets (N = 45)	CM (N = 47)	*P* value	RR (CI 95%)
Median (range)	3.3 (2.3-4.0)	1.3 (1.0-2.4)	**<.001**	
Score category, n (%)				
≤3	12 (26.7)	47 (100)	**<.001**	
>3	33 (73.3)	0 (0)		Not calculated

*Abbreviations:* CM, comfort maker.

Median was calculated on the mean scores attributed by the 3 observers for each patient. Calculation of the relative risk (RR) was not possible.

Comparing the scores at the end of RT versus after 6 months, the median score on the latter was significantly higher both in patients receiving lancets (median of 2.9 vs 3.3, respectively; *P*=<.001) and CM (median of 1.2 vs 1.3, respectively; *P*=<.001). The photographs of 2 participants, one from each arm, are provided in the Supplementary Appendix (Fig. E4).

### Patients’ satisfaction

Patients receiving lancets and CM had no significant difference in the satisfaction scores (Table 4), with a median score of 10 for both arms (*P*=.952). Four patients (4.4%) attributed a score below 6 (2 for each arm, *P*=1.000), 3 of which justified that with “tattoos too visible” (1 received lancet and 2 received CM) and 1 with “tattoos too blurred” (the patient received lancet). The distribution of the patients’ satisfaction scores is provided in the Supplementary Appendix (Fig. E5).

**Table 4:** Transversal analysis of patients’ satisfaction by study arm

Variable	Lancets (N = 44)	CM (N = 47)	*P* value	RR (CI 95%)
Median (range)	10 (5-10)	10 (1-10)	.952	
Score category:				
≤6	2 (4.4)	2 (4.3)	1.000	
>6	43 (95.6)	45 (95.7)		1.00 (95% CI, 0.92-1.09)*

*Abbreviations:* CM, comfort maker; RR, relative risk.

Lancet was the reference group when the RR was calculated.

⁎ RR for satisfaction >6.

## Discussion

We demonstrated that, 6 months after the end of RT, both methods used to tattoo the set-up markings (the CM or the disposable lancets) have a different profile regarding cosmesis and fading, with similar results concerning patients’ satisfaction.

On the photographic assessment, the CM had better cosmetic results while worst fading scores. Concerning the cosmesis evaluation, the CM group had a median score significantly higher. Furthermore, we found that regardless of the method used, the tattoos retain their cosmetic appearance in these first months after RT. On the fading assessment, a superiority in favor of the lancets was noticeable. Unlike the cosmesis assessment, the fading is a dynamic process, as either method had higher fading scores 6 months after finishing the RT compared with the end of the treatments.

We observed both methods produce markings that are characteristically unalike in their appearance, which translated by the significantly different scores attributed by the evaluators. The lancets tend to produce a more blurred and greenish marking, that although less cosmetically pleasant, tend to fade more over time. The CM tattoos, on the other hand, tend to keep round and with well-defined edges, keeping the heavy pigmentation unchanged over time, with only a minimal fading registered. These differences in cosmesis and fading might be explained by the distinct needle insertion depth and, this way, by the phagocytic response on the skin.^6^ The lancet needle injects the pigment with variable depths from 1 to 4 mm, so it affects the epidermis and the top layer of the dermis. The CM pricks only 0.2 mm, hence affecting the epidermis alone.^7^ The deeper the pigment is applied, the more expressive the migration of phagocytes to the dermis is, which might explain the superior fading observed with the deeper pigment injections the lancets produce.

Despite these results, none of the tattooing methods were associated with higher satisfaction scores by the patients.

Despite the clear advantages of dark-ink tattoos, they are associated with limitations. It is acknowledged that an acceptable cosmetic outcome is important.^8^ Tattoos are a physical tie to an emotional and difficult time in life and can lead to psychological challenges.^9^ In a survey applied to breast cancer patients treated with RT, around 70% of women had negative feelings about having permanent tattoos.^10^ This emphasizes the need for, when set-up markings are necessary, methods that produce cosmetically better markings, so to ameliorate the body image dissatisfaction and improve the patient experience.

The issue of patients' displeasure at being permanently marked is also well-recognized.^8^^,^^11^ To address the permanent issues with dark ink tattoos, there has been research into alternatives. This includes semipermanent methods, comprising henna ink, temporary tattoo seals, and oil-based pens.^12^, ^13^, ^14^, ^15^ Although they allow for less painful markings, these methods are inferior to dark-ink markings in terms of patient comfort, durability, and longevity.^8^^,^^12^ Another option is using ultraviolet ink. It allows the tattoo to become undetectable in ambient lighting, without compromising the setup accuracy.^16^^,^^17^

COMFORTATTOO is the first randomized trial to compare 2 permanent marking systems, which limits the comparison with our results. Most papers available focus on the use of semipermanent methods.^12^, ^13^, ^14^, ^15^ One study compares semipermanent to permanent marking. On the assessment of patients’ satisfaction with body image, the authors report dark-ink permanent markings impose a cosmetic problem for patients, which translates into a benefit for the nonpermanent methods.^12^ Another study compared fluorescent-ink and dark-ink tattoos, and on the evaluation regarding differences in body perception, fluorescent-ink were associated with increased body image satisfaction scores.^16^ The results of both these studies cannot be directly compared with ours, as none rely on an electric marking device, which has different implications in terms of cosmesis, fading and satisfaction than the historic lancets, so further investigation using an electric marking device CM is needed. More recently, there have been emerging trends toward tattoo-less setup by using surface guidance motion management tools, delivering at least equivalent accuracy in patient positioning.^3^^,^^18^^,^^19^ However, for centers that are not equipped, yet, with these systems, the skin markings remain essential for set-up.

## Conclusion

When evaluated 6 months after finishing the RT, tattooing the set-up markings with the Comfort Maker 2.0 produces cosmetically better markings however with less fading, compared with the lancets. Although, no method is associated with higher patients’ satisfaction scores.

## Disclosures

The authors declare that they have no other known competing financial interests or personal relationships that could have appeared to influence the work reported in this paper.

## Appendix Supplementary materials

Supplementary Appendix 6m Image, application 1

## Footnotes

Supplementary material associated with this article can be found in the online version at doi:10.1016/j.adro.2023.101404.
