# Supplementary material for: Radiation Therapy Skin Marking with Lancets Versus Electric Marking Pen (COMFORTATTOO)—6 Months Results on Cosmesis, Fading, and Patients’ Satisfaction From a Randomized, Double-Blind Trial
Source: Adv Radiat Oncol. 2023 Nov 5;9(3):101404. doi: 10.1016/j.adro.2023.101404 (PMC10823085; doi:10.1016/j.adro.2023.101404)
Supplement: Supplementary Appendix 6m [file mmc1.docx]

**Supplementary Appendix**

**Figure S1:** 5-scale fading scores used in the fading assessment.

**Figure S2:** Histogram of the frequency distribution of the mean scores attributed by the 20 observers on the photographic cosmesis assessment 6 months after finishing radiotherapy.

**Figure S3:** Histogram of the frequency distribution of the mean scores attributed by the 3 observers on the photographic fading assessment 6 months after finishing radiotherapy.

**Figure S4.** Individual photographs of the set-up markings recorded 6 months after the end of RT

**
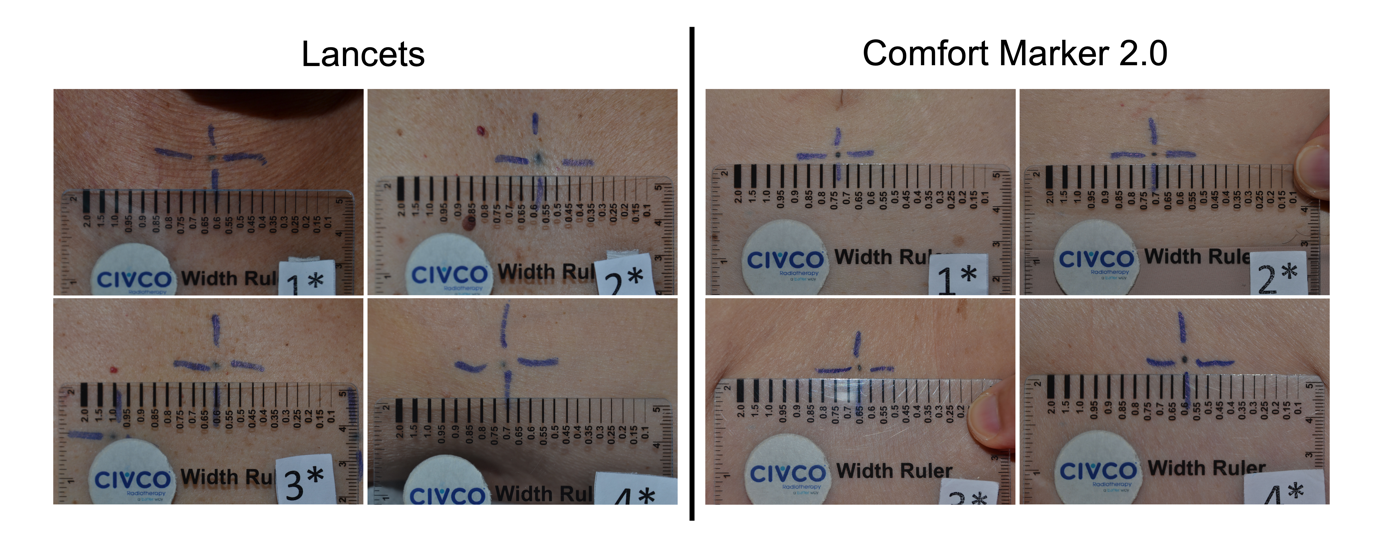
**

**Figure S5:** Histogram of the frequency distribution of the patients’ satisfaction scores 6 months after finishing radiotherapy.
